# Supplementary material for: A DNA Damage Response Gene Panel for Different Histologic Types of Epithelial Ovarian Carcinomas and Their Outcomes
Source: Biomedicines. 2021 Oct 3;9(10):1384. doi: 10.3390/biomedicines9101384 (PMC8533221; doi:10.3390/biomedicines9101384)
Supplement: Supplementary file 1 [file biomedicines-09-01384-s001.zip › OV NGS Table S1.pdf]

Table S1: Variants of uncertain significance (VUS) with the potential of being deleterious mutations in all patients

Gene	Transcript	cDNA position	Amino acid	ESP6500	1000 Genomics	SIFT	Polyphen2	rs number
ATM	NM_000051	c.C6934T	p.L2312F	NA	NA	0	1	NA
ATM	NM_000051	c.G2781T	p.L927F	NA	NA	0.01	1	NA
ATM	NM_000051	c.G902A	p.G301D	0.000077	NA	0.002	0.999	rs202208861
ATM	NM_000051	c.G8608A	p.D2870N	NA	NA	0	1	rs55798854
ATM	NM_000051	c.A8450G	p.Y2817C	NA	NA	0.004	1	rs747764678
BARD1	NM_000465	c.C1972T	p.R658C	0.00592	0.005391	0.03	0.995	rs3738888
BARD1	NM_000465	c.A1298G	p.H433R	NA	NA	0.01	0.992	NA
BARD1	NM_000465	c.A1988G	p.N663S	NA	0.0002	0	1	rs187590361
ERCC5	NM_001204425	c.C2518T	p.R840W	NA	NA	0.002	0.994	rs758046666
ERCC5	NM_001204425	c.G4619A	p.G1540E	NA	NA	0	0.99	NA
BRCA1	NM_007294	c.G1033T	p.D345Y	NA	NA	0.01	0.993	rs80356961
BRCA1	NM_007294	c.C5501T	p.T1834I	NA	NA	0	0.999	NA
BRCA1	NM_007294	c.C3448T	p.P1150S	NA	0.000799	0.01	1	rs80357272
BRCA2	NM_000059	c.G7897T	p.A2633S	NA	NA	0.039	0.986	rs431825359
BRCA2	NM_000059	c.A8602G	p.K2868E	NA	NA	0.001	0.998	NA
BRCA2	NM_000059	c.C9872T	p.S3291F	NA	NA	0	1	NA
BRIP1	NM_032043	c.A1G	p.M1V	NA	NA	0	0.982	rs764585550
BRIP1	NM_032043	c.C2563T	p.R855C	0.000154	NA	0.001	0.999	rs146031731

CHEK2	NM_007194	c.C1561T	p.R521W	NA	0.0002	0.002	1	rs533475838
CHEK2	NM_007194	c.G1421A	p.R474H	0.000268	NA	0	1	rs121908706
CHEK2	NM_007194	c.G280A	p.A94T	NA	NA	0.005	0.964	rs753860983
ERCC2	NM_000400	c.A1256G	p.E419G	NA	NA	0	0.998	rs775219805
ERCC3	NM_000122	c.G1468A	p.E490K	0.000077	NA	0	0.994	rs367552856
ERCC6	NM_000124	c.G1801A	p.G601S	0.000231	0.000599	0.007	1	rs138758064
ERCC6	NM_000124	c.G385A	p.V129M	0.000154	0.001398	0.003	0.002	rs116360202
ERCC6	NM_000124	c.G1801A	p.G601S	0.000231	0.000599	0.007	1	rs138758064
ERCC6	NM_000124	c.G230A	p.R77Q	NA	NA	0.021	1	NA
FANCA	NM_000135	c.C334G	p.L112V	NA	NA	0	0.999	NA
FANCA	NM_000135	c.C4225T	p.R1409W	0.000077	0.0002	0.01	0.999	rs139478274
FANCA	NM_000135	c.G538A	p.E180K	NA	NA	0.004	0.991	NA
FANCA	NM_000135	c.C4225T	p.R1409W	0.000077	0.0002	0.005	0.999	rs139478274
FANCA	NM_000135	c.G2923A	p.G975R	0.000077	NA	0	1	rs369565161
FANCA	NM_000135	c.C2525A	p.S842Y	NA	NA	0.001	1	NA
FANCC	NM_000136	c.C1000T	p.R334W	0.000077	NA	0.002	0.99	rs140348260
FANCI	NM_001113378	c.C1261T	p.L421F	NA	NA	0.005	1	NA
FANCI	NM_001113378	c.G1741A	p.E581K	NA	NA	0.009	0.998	rs768875486
FANCM	NM_020937	c.G5026A	p.E1676K	NA	NA	0.01	1	NA
FANCM	NM_020937	c.C2996T	p.P999L	0.000692	0.000799	0	1	rs148304968
FANCM	NM_020937	c.A2852G	p.D951G	NA	NA	0.007	0.958	rs760309916
MLH3	NM_001040108	c.G890A	p.R297Q	0.000077	NA	0.03	1	rs374212925

MRE11	**NM_005590**	**c.G1724A**	**p.G575D**	**NA**	**NA**	**0.015**	**0.989**	**rs376555330**
MSH2	**NM_000251**	**c.G232A**	**p.V78I**	**NA**	**NA**	**0.03**	**0.998**	**NA**
MSH2	**NM_000251**	**c.G2197A**	**p.A733T**	**NA**	**NA**	**0**	**0.995**	**NA**
MSH2	**NM_000251**	**c.G1601T**	**p.R534L**	**NA**	**NA**	**0**	**1**	**NA**
MSH3	**NM_002439**	**c.G2913C**	**p.L971F**	**NA**	**NA**	**0**	**1**	**rs775386491**
MSH3	**NM_002439**	**c.G2913C**	**p.L971F**	**NA**	**NA**	**0**	**1**	**rs775386491**
MSH3	**NM_002439**	**c.C2335T**	**p.R779C**	**0.000154**	**NA**	**0**	**1**	**rs368162490**
MSH3	**NM_002439**	**c.G1366A**	**p.E456K**	**NA**	**NA**	**0.001**	**1**	**rs963234468**
MSH6	**NM_001281492**	**c.C2870A**	**p.P957H**	**0.000154**	**NA**	**0**	**0.998**	**rs63750753**
MSH6	**NM_001281492**	**c.C3266T**	**p.T1089I**	**NA**	**NA**	**0**	**1**	**rs63750949**
MSH6	**NM_001281492**	**c.C1910G**	**p.T637S**	**NA**	**NA**	**0.009**	**0.995**	**rs587781462**
MSH6	**NM_001281492**	**c.C1910G**	**p.T637S**	**NA**	**NA**	**0.009**	**0.995**	**rs587781462**
PALB2	**NM_024675**	**c.C109T**	**p.R37C**	**NA**	**NA**	**0**	**1**	**rs200048921**
PALB2	**NM_024675**	**c.G3054C**	**p.E1018D**	**NA**	**0.000799**	**0.02**	**0.998**	**rs183489969**
PMS1	**NM_000534**	**c.C287G**	**p.A96G**	**NA**	**NA**	**0**	**0.998**	**rs139414606**
PMS2	**NM_000535**	**c.C883T**	**p.R295W**	**NA**	**0.0002**	**0.001**	**1**	**rs182246929**
PMS2	**NM_000535**	**c.C883T**	**p.R295W**	**NA**	**0.0002**	**0.001**	**1**	**rs182246929**
POLD1	**NM_002691**	**c.A2602T**	**p.I868F**	**NA**	**NA**	**0**	**0.999**	**NA**
POLD1	**NM_002691**	**c.G2275A**	**p.V759I**	**0.001307**	**0.000998**	**0**	**1**	**rs145473716**
POLD1	**NM_002691**	**c.G2758A**	**p.D920N**	**NA**	**NA**	**0.001**	**0.974**	**rs1060501811**
POLE	**NM_006231**	**c.G2503A**	**p.V835I**	**NA**	**NA**	**0.01**	**1**	**NA**
POLE	**NM_006231**	**c.G3176A**	**p.R1059H**	**NA**	**NA**	**0**	**1**	**rs751425952**

POLE	**NM_006231**	**c.C833T**	**p.T278M**	**NA**	**NA**	**0.001**	**1**	**rs764254754**
POLE	**NM_006231**	**c.A712T**	**p.I238F**	**NA**	**0.0002**	**0.001**	**1**	**rs536684123**
POLE	**NM_006231**	**c.C857G**	**p.P286R**	**NA**	**NA**	**0**	**1**	**rs1057519943**
POLH	**NM_006502**	**c.C1433T**	**p.T478M**	**0.000077**	**0.005791**	**0.015**	**0.996**	**rs9296419**
POLH	**NM_006502**	**c.A40G**	**p.M14V**	**NA**	**0.0002**	**0**	**1**	**rs61748656**
POLH	**NM_006502**	**c.C1433T**	**p.T478M**	**0.000077**	**0.005791**	**0.015**	**0.996**	**rs9296419**
POLH	**NM_006502**	**c.C1433T**	**p.T478M**	**0.000077**	**0.005791**	**0.015**	**0.996**	**rs9296419**
POLH	**NM_006502**	**c.C1433T**	**p.T478M**	**0.000077**	**0.005791**	**0.015**	**0.996**	**rs9296419**
POLH	**NM_006502**	**c.G246T**	**p.E82D**	**NA**	**0.0002**	**0.024**	**0.999**	**rs77759052**
POLK	**NM_016218**	**c.C1048G**	**p.P350A**	**NA**	**0.000599**	**0.04**	**1**	**NA**
POLK	**NM_016218**	**c.A454G**	**p.M152V**	**NA**	**NA**	**0**	**0.999**	**NA**
POLK	**NM_016218**	**c.C1048G**	**p.P350A**	**NA**	**0.000599**	**0**	**0.998**	**rs528839357**
POLK	**NM_016218**	**c.C1048G**	**p.P350A**	**NA**	**0.000599**	**0**	**0.998**	**rs528839357**
POLK	**NM_016218**	**c.C892T**	**p.R298C**	**NA**	**NA**	**0**	**1**	**rs200548318**
POLK	**NM_016218**	**c.C1406T**	**p.T469I**	**NA**	**NA**	**0.004**	**0.999**	**NA**
RAD50	**NM_005732**	**c.C3790T**	**p.L1264F**	**NA**	**0.000399**	**0**	**1**	**rs199579239**
SLX4	**NM_032444**	**c.C1271T**	**p.A424V**	**NA**	**0.0002**	**0**	**1**	**NA**
SLX4	**NM_032444**	**c.G2449C**	**p.E817Q**	**0.000077**	**0.0002**	**0.03**	**0.999**	**rs143730668**
SLX4	**NM_032444**	**c.C4765T**	**p.R1589C**	**NA**	**0.000799**	**0**	**1**	**rs181782315**
SLX4	**NM_032444**	**c.C1271T**	**p.A424V**	**NA**	**0.0002**	**0**	**1**	**NA**
SLX4	**NM_032444**	**c.G2449C**	**p.E817Q**	**0.000077**	**0.0002**	**0.03**	**0.999**	**rs143730668**
SLX4	**NM_032444**	**c.C5383T**	**p.R1795C**	**NA**	**NA**	**0.004**	**0.98**	**rs762619200**

SLX4	**NM_032444**	**c.C5156A**	**p.S1719Y**	**NA**	**NA**	**0.001**	**1**	**NA**
TP53	**NM_000546**	**c.A707G**	**p.Y236C**	**NA**	**NA**	**0**	**1**	**NA**
TP53	**NM_000546**	**c.G713A**	**p.C238Y**	**NA**	**NA**	**0**	**1**	**NA**
TP53	**NM_000546**	**c.G469T**	**p.V157F**	**NA**	**NA**	**0.01**	**1**	**rs121912654**
TP53	**NM_000546**	**c.A536G**	**p.H179R**	**NA**	**NA**	**0**	**1**	**NA**
TP53	**NM_000546**	**c.C726G**	**p.C242W**	**NA**	**NA**	**0**	**1**	**NA**
TP53	**NM_000546**	**c.A757C**	**p.T253P**	**NA**	**NA**	**0**	**1**	**NA**
TP53	**NM_000546**	**c.A838G**	**p.R280G**	**NA**	**NA**	**0**	**1**	**NA**
TP53	**NM_000546**	**c.C535A**	**p.H179N**	**NA**	**NA**	**0**	**1**	**NA**
TP53	**NM_000546**	**c.T810G**	**p.F270L**	**NA**	**NA**	**0**	**1**	**NA**
TP53	**NM_000546**	**c.A578G**	**p.H193R**	**NA**	**NA**	**0.01**	**1**	**NA**
TP53	**NM_000546**	**c.A578T**	**p.H193L**	**NA**	**NA**	**0**	**1**	**NA**
TP53	**NM_000546**	**c.A659G**	**p.Y220C**	**NA**	**NA**	**0**	**1**	**NA**
TP53	**NM_000546**	**c.G796A**	**p.G266R**	**NA**	**NA**	**0**	**1**	**NA**
TP53	**NM_000546**	**c.C586G**	**p.R196G**	**NA**	**NA**	**0**	**1**	**NA**
TP53	**NM_000546**	**c.A631G**	**p.T211A**	**NA**	**NA**	**0**	**0.978**	**NA**
TP53	**NM_000546**	**c.A707G**	**p.Y236C**	**NA**	**NA**	**0**	**1**	**NA**
TP53	**NM_000546**	**c.T584C**	**p.I195T**	**NA**	**NA**	**0**	**1**	**NA**
TP53	**NM_000546**	**c.A740T**	**p.N247I**	**NA**	**NA**	**0**	**1**	**NA**
TP53	**NM_000546**	**c.A578G**	**p.H193R**	**NA**	**NA**	**0.01**	**1**	**NA**
TP53	**NM_000546**	**c.A536G**	**p.H179R**	**NA**	**NA**	**0**	**0.989**	**rs1057519991**
TP53	**NM_000546**	**c.T470A**	**p.V157D**	**NA**	**NA**	**0**	**1**	**NA**

XPA	**NM_000380**	**c.C772T**	**p.R258C**	**NA**	**0.000399**	**0.03**	**0.983**	**rs188860873**
XPC	**NM_004628**	**c.C586T**	**p.R196C**	**NA**	**NA**	**0.001**	**1**	**NA**
XRCC3	**NM_005432**	**c.G662A**	**p.C221Y**	**NA**	**0.0002**	**0.023**	**0.99**	**rs201886982**
