# Supplementary material for: A DNA Damage Response Gene Panel for Different Histologic Types of Epithelial Ovarian Carcinomas and Their Outcomes
Source: Biomedicines. 2021 Oct 3;9(10):1384. doi: 10.3390/biomedicines9101384 (PMC8533221; doi:10.3390/biomedicines9101384)
Supplement: Supplementary file 1 [file biomedicines-09-01384-s001.zip › OV NGS Table S2.pdf]

Table S2-1: The percentages of DDR genes mutations in all patients

Histology	ALL	Serous carcinoma	Endometrioid carcinoma	Clear cell carcinoma
**Wild type**	54.70%	30.40%	66.70%	73.40%
BARD1	0.6%	0.0%	0.0%	1.6%
BRCA1	1.2%	1.4%	0.0%	1.6%
BRCA2	4.1%	5.8%	2.6%	3.1%
BRIP1	0.6%	1.4%	0.0%	0.0%
FANCC	0.6%	0.0%	2.6%	0.0%
FANCG	0.6%	1.4%	0.0%	0.0%
FANCI	0.6%	0.0%	0.0%	1.6%
FANCM	0.6%	0.0%	2.6%	0.0%
MRE11	0.6%	0.0%	2.6%	0.0%
RAD50	1.7%	0.0%	5.1%	1.6%
RAD51C	2.3%	5.8%	0.0%	0.0%
RAD51D	0.6%	1.4%	0.0%	0.0%
MLH1	1.2%	0.0%	2.6%	1.6%
MLH3	2.3%	1.4%	5.1%	1.6%
MSH2	1.7%	0.0%	7.7%	0.0%
MSH3	0.6%	0.0%	2.6%	0.0%
MSH6	1.7%	1.4%	2.6%	1.6%
MUTYH	6.4%	4.3%	5.1%	9.4%
OGG1	0.6%	1.4%	0.0%	0.0%
XRCC4	0.6%	0.0%	0.0%	1.6%
XRCC6	0.6%	1.4%	0.0%	0.0%
ERCC8	2.9%	4.3%	0.0%	3.1%
POLD1	0.6%	0.0%	0.0%	1.6%
ATM	3.5%	1.4%	12.8%	0.0%
CHEK2	0.6%	0.0%	0.0%	1.6%
TP53	27.9%	56.5%	15.4%	4.7%

Table S2-2: The percentages of DDR genes mutations by subgroups

Histology	ALL	Serous carcinoma	Endometrioid carcinoma	Clear cell carcinoma
**Wild type**	54.70%	30.40%	66.70%	73.40%
**HR**	10.5%	15.9%	7.7%	6.3%
**NHEJ**	1.2%	1.4%	0.0%	1.6%
**MMR**	6.4%	2.9%	15.4%	4.7%
**BER**	7.0%	5.8%	5.1%	9.4%
**NER**	2.9%	4.3%	0.0%	3.1%
**TLS**	0.6%	0.0%	0.0%	1.6%
**CCR**	30.8%	58.0%	23.1%	6.3%
**DDR**	45.3%	69.6%	33.3%	26.6%
