# Supplementary material for: A DNA Damage Response Gene Panel for Different Histologic Types of Epithelial Ovarian Carcinomas and Their Outcomes
Source: Biomedicines. 2021 Oct 3;9(10):1384. doi: 10.3390/biomedicines9101384 (PMC8533221; doi:10.3390/biomedicines9101384)
Supplement: Supplementary file 1 [file biomedicines-09-01384-s001.zip › OV NGS Table S3.pdf]

Table S3. The percentages of subgroups of DDR gene mutations by clinical factors in the patients

Genes		Histology			FIGO stage		Tumor grade		Recurrence		Death	
		OSA	OEA	OCCA	Early	Advanced	Low	High	No	Yes	No	Yes
	172	69	39	64	69	103	29	143	70	102	96	76
**HR**												
**Wild type**	154	58	36	60	64	90	26	128	64	90	89	65
	89.5%	84.1%	92.3%	93.8%	92.8%	87.4%	89.7%	89.5%	91.4%	88.2%	92.7%	85.5%
**Mutation**	18	11	3	4	5	13	3	15	6	12	7	11
	10.5%	15.9%	7.7%	6.3%	7.2%	12.6%	10.3%	10.5%	8.6%	11.8%	7.3%	14.5%
**P value***				0.154		0.259		0.981		0.502		0.126
**NHEJ**												
**Wild type**	170	68	39	63	69	101	29	141	70	100	96	74
	98.8%	98.6%	100.0%	98.4%	100.0%	98.1%	100.0%	98.6%	100.0%	98.0%	100.0%	97.4%
**Mutation**	2	1	0	1	0	2	0	2	0	2	0	2
	1.2%	1.4%	0.0%	1.6%	0.0%	1.9%	0.0%	1.4%	0.0%	2.0%	0.0%	2.6%
**P value***				0.742		0.244		0.522		0.239		0.11
**MMR**												
**Wild type**	161	67	33	61	65	96	24	137	66	95	91	70
	93.6%	97.1%	84.6%	95.3%	94.2%	93.2%	82.8%	95.8%	94.3%	93.1%	94.8%	92.1%
**Mutation**	11	2	6	3	4	7	5	6	4	7	5	6
	6.4%	2.9%	15.4%	4.7%	5.8%	6.8%	17.2%	4.2%	5.7%	6.9%	5.2%	7.9%

**P value***				0.03		0.793		0.009		0.762		0.475
**BER**												
**Wild type**	160	65	37	58	65	95	27	133	66	94	91	69
	93.0%	94.2%	94.9%	90.6%	94.2%	92.2%	93.1%	93.0%	94.3%	92.2%	94.8%	90.8%
**Mutation**	12	4	2	6	4	8	2	10	4	8	5	7
	7.0%	5.8%	5.1%	9.4%	5.8%	7.8%	6.9%	7.0%	5.7%	7.8%	5.2%	9.2%
**P value***				0.631		0.619		0.985		0.59		0.306
**NER**												
**Wild type**	167	66	39	62	67	100	29	138	67	100	93	74
	97.1%	95.7%	100.0%	96.9%	97.1%	97.1%	100.0%	96.5%	95.7%	98.0%	96.9%	97.4%
**Mutation**	5	3	0	2	2	3	0	5	3	2	3	2
	2.9%	4.3%	0.0%	3.1%	2.9%	2.9%	0.0%	3.5%	4.3%	2.0%	3.1%	2.6%
**P value***				0.43		0.996		0.307		0.373		0.848
**TLS**												
**Wild type**	171	69	39	63	69	102	29	142	70	101	96	75
	99.4%	100.0%	100.0%	98.4%	100.0%	99.0%	100.0%	99.3%	100.0%	99.0%	100.0%	98.7%
**Mutation**	1	0	0	1	0	1	0	1	0	1	0	1
	0.6%	0.0%	0.0%	1.6%	0.0%	1.0%	0.0%	0.7%	0.0%	1.0%	0.0%	1.3%
**P value***				0.428		0.412		0.652		0.406		0.26
**CCR**												
**Wild type**	119	29	30	60	60	59	24	95	57	62	74	45
	69.2%	42.0%	76.9%	93.8%	87.0%	57.3%	82.8%	66.4%	81.4%	60.8%	77.1%	59.2%

**Mutation**	53	40	9	4	9	44	5	48	13	40	22	31
	30.8%	58.0%	23.1%	6.3%	13.0%	42.7%	17.2%	33.6%	18.6%	39.2%	22.9%	40.8%
**P value***				<0.001		<0.001		0.083		0.004		0.012

Note: OSA: ovarian serous carcinoma; OEA: ovarian endometrioid carcinoma; OCCA: ovarian clear cell carcinoma; HR: homologous recombination; NHEJ: nonhomologous DNA end joining; MMR: mismatch repair; BER: base excision repair; NER: nucleotide excision repair; TLS: translesion synthesis; CCR: cell cycle regulation;*Pearson's chi-squared test
