# Supplementary figures and images for: A DNA Damage Response Gene Panel for Different Histologic Types of Epithelial Ovarian Carcinomas and Their Outcomes
Source: Biomedicines. 2021 Oct 3;9(10):1384. doi: 10.3390/biomedicines9101384 (PMC8533221; doi:10.3390/biomedicines9101384)

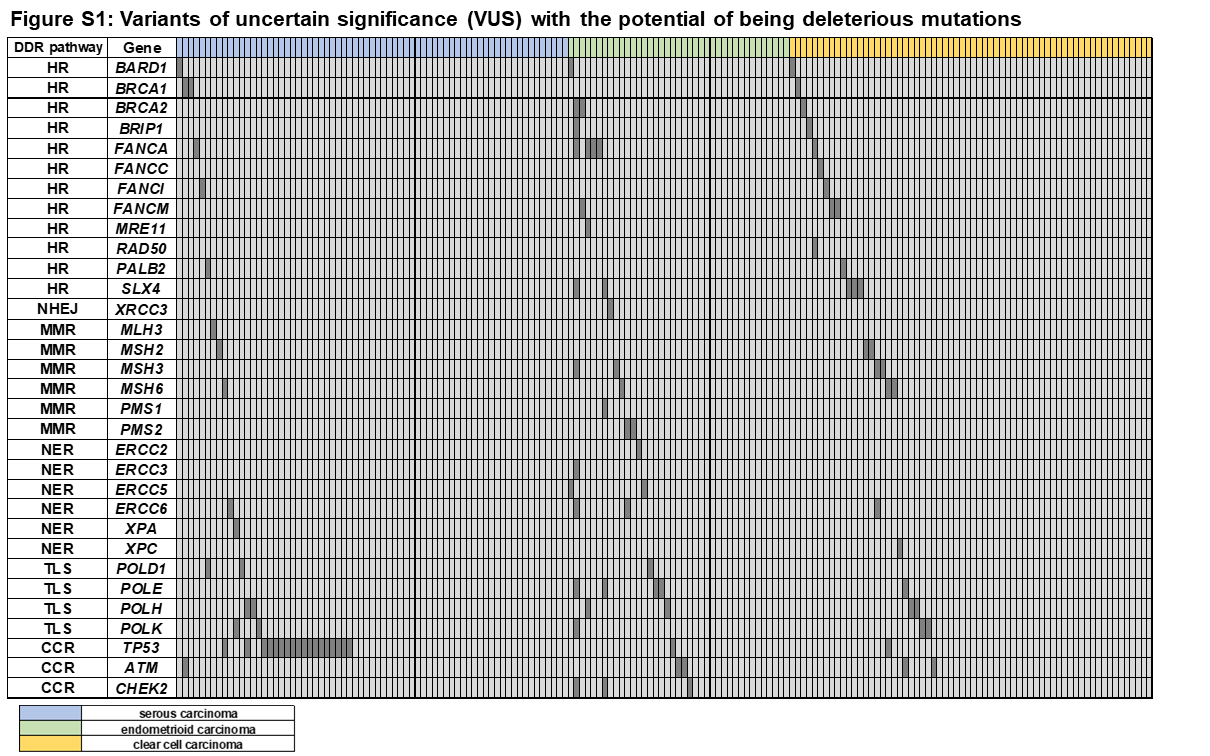

Supplement: Supplementary file 1 [file biomedicines-09-01384-s001.zip › OV NGS Figure S1.tif]

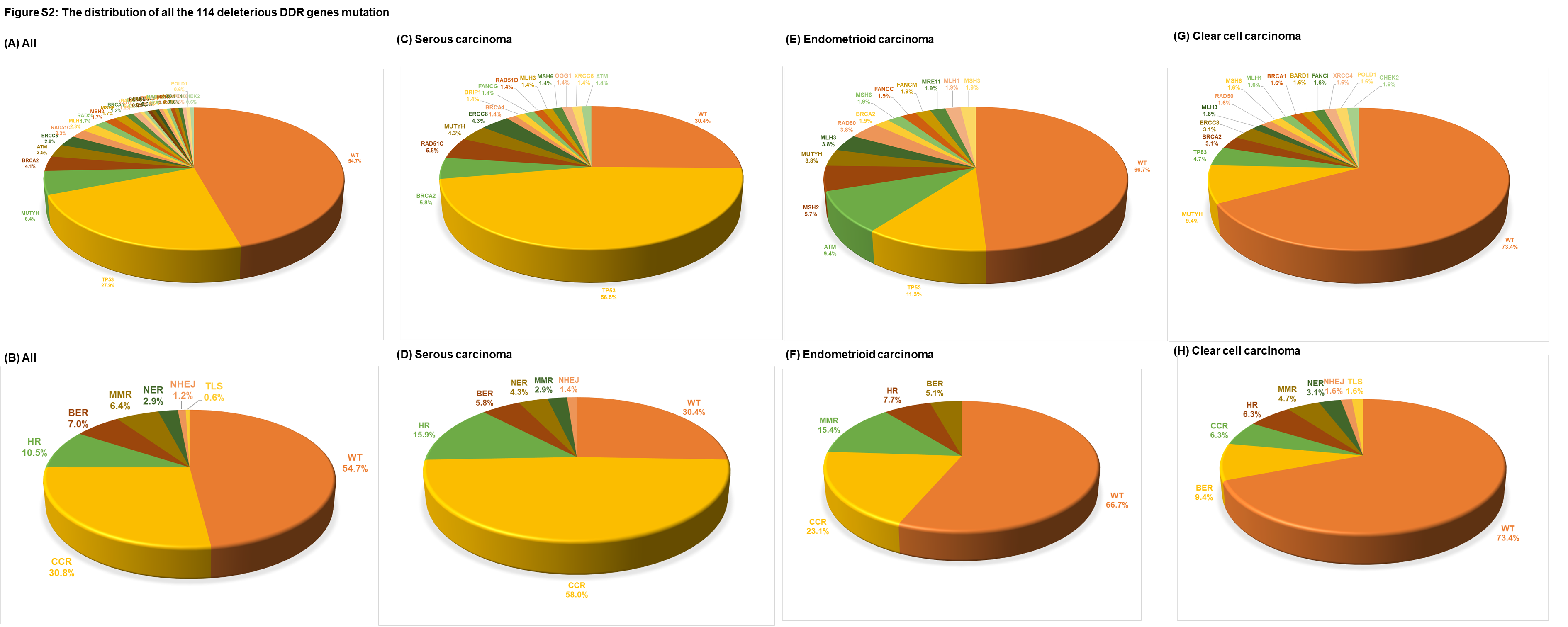

Supplement: Supplementary file 1 [file biomedicines-09-01384-s001.zip › OV NGS Figure S2.tif]
